# A Review on the Relationship between Tocotrienol and Alzheimer Disease

**DOI:** 10.3390/nu10070881

**Published:** 2018-07-09

**Authors:** Kok-Yong Chin, Shu Shen Tay

**Affiliations:** 1 Department of Pharmacology, Faculty of Medicine, Universiti Kebangsaan Malaysia, Cheras 56000, Malaysia; 2 Columbia Asia Hospital, Miri 98009, Malaysia; sstay87@gmail.com

**Keywords:** brain, cognition, memory, mitochondria, neuron, oxidative stress, vitamin E

## Abstract

Alzheimer’s disease (AD) is plaguing the aging population worldwide due to its tremendous health care and socioeconomic burden. Current treatment of AD only offers symptomatic relief to patients. Development of agents targeting specific pathologies of AD is very slow. Tocotrienol, a member of the vitamin E family, can tackle many aspects of AD, such as oxidative stress, mitochondrial dysfunction and abnormal cholesterol synthesis. This review summarizes the current evidence on the role of tocotrienol as a neuroprotective agent. Preclinical studies showed that tocotrienol could reduce oxidative stress by acting as a free-radical scavenger and promoter of mitochondrial function and cellular repair. It also prevented glutamate-induced neurotoxicity in the cells. Human epidemiological studies showed a significant inverse relationship between tocotrienol levels and the occurrence of AD. However, there is no clinical trial to support the claim that tocotrienol can delay or prevent the onset of AD. As a conclusion, tocotrienol has the potential to be developed as an AD-preventing agent but further studies are required to validate its efficacy in humans.

## 1. Introduction

Alzheimer’s disease (AD) is a spectrum of neurodegenerative illnesses defined by clinical symptoms and biological changes in the brain. The characteristic clinical symptoms of AD include progressive dementia with episodic memory impairment. The biological changes refer to the presence of neurofibrillary entanglement, senile plaques, amyloid deposition and synaptic loss in the brain. Since the neurobiological changes cannot be detected until post-mortem, diagnosis based on clinical symptoms alone is probabilistic and refers to as probable AD [1]. However, with the progress in medical technology, in vivo detection of pathological changes in the brain directly via advanced imaging techniques (magnetic resonance imaging (MRI) and positron emission tomography (PET)) and indirectly via biomarkers in cerebrospinal fluid (CSF) (tau, phosphorylated tau and 42 amino acid isoform of beta-amyloid protein (Aβ42)), is made possible nowadays [2,3]. The combination of neuropsychological scores, neuroimaging and CSF biomarkers offers enhanced prediction on the development of AD in patients with mild cognitive impairment [4].

Apart from memory loss, AD also erodes the cognitive function of patients, subsequently affecting their ability to self-care. Considering the exponential increase in the elderly population worldwide, especially those age 65 years and above, the healthcare and financial burden of AD is tremendous. An estimate performed in 2010 indicated that 4.7% of the world population aged 65 years and above are affected by dementia and AD, with the highest prevalence in Europe and North America [5]. In fact, it is the fifth leading cause of death for the elderly in the United States [6]. By 2050, AD is projected to cause 1.6 million deaths in elderly Americans [7]. However, the greatest increment in the prevalence of AD will occur in middle-income countries in Africa and Asia (370% and 282% in 2050 compared to 2010) [5]. In terms of economic burden, AD and dementia incurred 604 billion USD direct medical and societal costs, as well as informal care in 2010, equivalent to 1% of the global gross domestic product [8]. By 2030, the cost will elevate by 85% [8]. In view of the colossal impacts of AD, enormous efforts have been invested to prevent or cure the illness. Delaying the onset/progression of AD for one year is estimated to prevent 9.2 million cases of death in 2050 [9].

The current treatments for AD are acetylcholinesterase inhibitors (donepezil, galantamine, rivastigmine) and N-methyl d-aspartate receptor antagonists (memantine) [10]. They can slow down the progression and relieve the symptoms of AD but cannot cure the disease [10]. Several disease-modifying agents for AD, for instance, anti-Aβ and anti-tau agents, are currently under study, but progress is slow [11]. Attention has turned to interventions to prevent or delay the onset of AD, including dietary ones. Vitamin E is one of the nutrients being extensively studied for its preventive effects on AD [12]. Vitamin E is classified into two major groups, i.e., tocopherols and tocotrienols. Each group is further divided to alpha-, beta-, gamma-, delta- homologues based on the position of the methyl side chains on the chromanol ring [13]. Both tocopherols and tocotrienols are present in a mixture in natural resources, such as plant-based oils (palm, annatto, rice bran) and grains (wheat, oat, barley), in varying composition [14]. Vitamin E is well-known for its antioxidative and anti-inflammatory properties, thus justifying its role in preventing oxidative stress and inflammation in the brain [14]. Tocopherols, especially alpha-tocopherol, have been the focus of previous research [12]. However, tocotrienols possess biological activities not found in tocopherols, notably its mevalonate pathway suppressive actions [15]. Tocotrienols also exhibit stronger antioxidant and anti-inflammatory activities compared to alpha-tocopherol [16]. This discrepancy arises due to two factors: (i) tocotrienols possess shorter side chains than alpha-tocopherol which allow them to be incorporated into the cell membrane better; (ii) alpha-tocopherol is preferentially retained by body tissues, but tocotrienols are rapidly degraded to short-chain carboxychromanols and conjugated counterparts, which might possess better biological effects [17]. Therefore, it could be more effective than alpha-tocopherol in preventing AD.

This review aims to summarize the current evidence of the effects of tocotrienols in AD. We searched two major databases (Scopus and Pubmed) using keywords “tocotrienols” AND “Alzheimer disease” OR “cognitive impairment” from 1 April 2018 to 30 April 2018. Original research articles written in English were retrieved. Two authors independently screened the articles, discussed and reached consensus on the inclusion of a study in this review. Evidence on cell culture, animal and human studies were included to present a comprehensive overview on the topic.

## 2. Pathogenesis of Alzheimer Disease

Development of a treatment for AD entails understanding of its pathogenesis process. Neuronal cell death due to aberrant protein processing is the major characteristic in the development of AD. Two hallmarks of AD are the presence of neurofibrillary tangles (NFTs) and senile plaques (SPs) in the brains [18]. Neurofibrillary tangles are intraneuronal protein deposits of hyperphosphorylated tau protein. Extracellular SPs, on the other hand, consist of neurotoxic oligomeric β-amyloid (Aβ) peptide clusters formed as a result of misprocessed amyloid precursor protein (APP) by β- and γ- secretases. Several theories detailing the pathological accumulation of NFTs and SPs in the brain have been put forward, notably the Aβ hypothesis [19], the mitochondrial cascade hypothesis [20] and the glutamate dysfunction hypothesis [21].

Cerebrovascular amyloid protein deposition in the AD brain was initially reported by Glenner and Wong [22]. Hardy and Higgins later proposed the amyloid cascade hypothesis to explain the causality of AD [19]. It gathered a considerable amount of clinical and laboratory evidence over the subsequent years [18,19]. While distinguished in the temporal sequence of underlying pathogenic events, the 42-residue Aβ (Aβ42) isoform appears as the common initiating factor and primary constituent in senile plaque during the development of both early-onset and late-onset AD. Progression of early-onset AD entails familial AD (FAD)-linked missense mutations in presenilins, the catalytic subunit of gamma-secretase. Mutated gamma-secretase increases the generation of highly self-aggregating Aβ42 isoform, consequently promoting its cerebral and peripheral deposition. Beyond the role of Aβ42 isoform, cell-based experiments have also disclosed pathogenic effect of FAD mutation ensuing from overall altered qualitative Aβ profile [23]. In contrast, decreased efficiency of Aβ clearance in the brain caused by the apolipoprotein E4 (ApoE4) isoform coding ε4 allele coupled with dysfunction ATP-binding cassette transporter A7 (ABCA7) is regarded as the major event leading to senile plaque deposition in late-onset AD [18,24]. Detrimental effects of ε4 allele extend to its coding product, ApoE4, which has a lower antioxidative capacity as compared to other ApoE isoforms, thus increasing the oxidative damage in neuronal cells [25]. Independent of plaque formation, Aβ42 also presents as a neurotoxic substance that gives rise to synaptic transmission deficits in the brains of mouse models [26].

High cholesterol level has been implicated in the development of AD. Cholesterol biosynthesis and elevated cholesterol level have been found to associate with increased proteolysis of the amyloid precursor protein (APP) by beta- and gamma-secretase to generate Aβ. Animal studies demonstrated elevation of Aβ load in the central nervous system of transgenic mouse and brain cortices of rabbit following chronic feeding of cholesterol-enriched diet [27]. Cholesterol-protein binding blot assay later revealed that direct binding of cholesterol to the alpha-secretase cleavage site of APP promotes beta-secretase activity and Aβ generation [28]. Cutler et al. (2004) have reported deviations in cholesterol metabolism that led to building up of cholesterol in the brains of AD patients [29]. Based on amyloid cascade theory, lowering of cholesterol levels could alleviate Aβ load in the brain. Thus, it can be a tantalizing AD prevention and treatment method. Nonetheless, the role of cholesterol, whether it is the cause or the consequence of AD, remains ambiguous [15]. Statins, a class of hypocholesterolemic agent, could reduce cholesterol biosynthesis and subsequently the Aβ generation via competitive inhibition of 3-hydroxy-3-methylglutaryl coenzyme A (HMG CoA) reductase, the rate-limiting enzyme in the mevalonate pathway. A meta-analysis of 20 observational studies showed that statin users had a lower relative risk (RR) of AD compared to non-users (RR: 0.70, 95% confidence interval (CI): 0.60–0.83) [30].

Despite extensive supporting evidence, weaknesses in the amyloid cascade hypothesis, notably the lack of success of Aβ-based interventions and the discordance between Aβ load and AD cognitive impairment, left the existence of the theory questionable [20,31]. It remains disputable whether amyloid accumulation plays a causal role in the pathogenesis of AD or if it is a consequence of other AD pathological mechanisms [18,32]. The mitochondrial cascade theory underpins the later and suggests that mitochondrial dysfunction provokes dysregulation of the amyloid cascade, including altered APP expression, increased APP processing, Aβ generation and accumulation [20]. On top of amyloid cascade events, the concomitant mitochondrial dysfunction is also consistent with AD-associated molecular events, particularly oxidative stress and insults, dysregulated tau phosphorylation and neuroinflammation [20]. This provides further support for the involvement of mitochondria in the pathogenesis of late-onset AD. Oxidative stress refers to an imbalance between production and detoxification of free radicals or reactive oxygen species (ROS). ROS is a group of physiological by-products responsible for the oxidization of major biomolecules, leading to cell damage and cell death [33]. Mitochondria are the main generator of ROS and are highly susceptible to oxidative damage. Interactive consequences of mitochondrial dysfunction and oxidative stress are amplified in the brain, which has high energy demand, high oxygen consumption, an abundance of easily peroxidizable polyunsaturated fatty acids, high level of potent ROS catalyst iron, and the relative paucity of antioxidants and related enzymes. As such, neuronal cells are especially vulnerable to oxidative stress-induced neurotoxicity associated with the development of AD [34].

The glutamate dysfunction hypothesis is one of the earliest proposed, yet substantial hypothesis in AD research [21]. Glutamate is a key excitatory neurotransmitter in mammalian central nervous system that plays a major role in memory formation and learning [35]. Unfortunately, the excitotoxic amino acid also has deleterious effects that are widely implicated in the development of AD [36,37]. Early studies have reported that excessive glutamate in the brain brings on overactivation of glutamate N-methyl-D-aspartate (NMDA) receptors, which causes the disruption of cellular energy metabolism, consequently energy inadequacy to maintain neuronal cell structures. Overactivation of NMDA receptors is also accompanied by overloading of intracellular calcium (Ca^2+^), resulting in neurodegeneration, subsequently cortical and subcortical atrophy [21,35]. Later, it has been found that Aβ production augments glutamate accumulation and then upregulates NMDA receptor activity. The activation of NMDA receptor further enhances Aβ production, forming a positive feedback loop that precedes NMDA receptor impairment and reduction, ultimately leading to neuronal cell death in AD brains [38]. Glutamate is also responsible for inducing antioxidative glutathione depletion, increasing ROS generation, and promoting membrane lipid oxidation, which are associated to the pathogenesis of AD [39,40].

## 3. Evidence from Cell Culture Studies

Cell-based studies have unambiguously demonstrated the efficiency of tocotrienols in alleviating oxidative stress, thus protecting neuronal cells from oxidative stress-induced cytotoxicity that leads to the development of AD. By employing neutral red cytotoxicity assay, Huebbe et al. (2007) showed that pre-treatment with 25 μM alpha-tocotrienol prior to three-hour exposure to organic peroxide, tert-Butyl hydroperoxide, exerted an absolute cytoprotective effect on SH-SY5Y human neuroblastoma cells. Similar pre-treatment also protected 50–60% SH-SY5Y cells from cytotoxicity upon exposure to H_2_O_2_ following depletion of glutathione, an important cellular antioxidant, by buthionine sulfoximine. Nonetheless, alpha-tocotrienol pre-treatment was futile against nitrosative stress induced by exposure to DETA/NO. The authors proposed the involvement of glutathione metabolism in mediating neuroprotective effects of tocotrienols based on previous studies but did not experimentally elucidate the underlying pathway or mechanism. With regards to the potency of vitamin E isoforms, their study showed that alpha-tocotrienol had the most potent neuroprotective properties against oxidative insults. However, considering the difference in cellular uptake of vitamin E isoforms and that the authors did not state the duration of tocotrienol pre-treatment in their article, neuroprotective potency of alpha-tocotrienol as compared with other vitamin E isoforms remains debatable [25].

Two later studies further furnished the evidence on antioxidative actions of tocotrienols in protecting neuronal cells against glutamate-induced cell death [41,42]. In one of the studies, Saito and colleagues (2010) compared the effects of pre-treatment and cotreatment with vitamin E isoforms on primary cortical neurons isolated from the cerebral cortex of Sprague-Dawley rat foetuses. Pre-treatment of cells with 0.25/2.5 μM alpha-/gamma-tocotrienols or alpha-/gamma-tocopherols for 24 h subsequently with addition of 10 mM glutamate for the next 24 h, or simultaneous incubation of both vitamin E isoforms and 10 mM glutamate for 24 h showed that tocotrienols diminished glutamate-induced cytotoxicity in both pre- and cotreatment, wherein the protective capacity of alpha-tocotrienol is higher than gamma-tocotrienol. The study delved into the antioxidative mechanism and found that at cytoprotective concentration, alpha-tocotrienol acted as a ROS scavenger rather than an inhibitor of ROS generation due to glutathione depletion. DPPP fluorescent probe detection also revealed that tocotrienols efficiently reduced lipid peroxidation in the cells. However, changes in the expression of GSH related genes and lipid peroxidation enzymes were not examined in this study [41]. In contrast to the results of Huebbe et al. (2007) [25], the authors reported that the cytoprotective capacities of vitamin E isoforms against glutamate challenge were similar but their efficiency depended on intracellular concentrations. This explained the larger effects of alpha-tocotrienol in the cell-based system to its higher incorporation rate into phospholipid bilayer membrane of cultured cells as compared to tocopherols. They suggested that alpha-tocopherol, which has greater cellular uptake and higher bioavailability, is a more potent neuroprotective agent than alpha-tocotrienol in vivo [41].

Complementary results were later observed in the second study carried out by Selvaraju et al. (2014) on the neuroprotective effects of the tocotrienol-rich fraction on astrocytes against glutamate-induced insults. Taking the uptake efficiency of both tocotrienol-rich fraction and alpha-tocopherol into account, DBTRG-05MG human glioblastoma cells were briefly pre-incubated for only 5 min with 100, 200, or 300 ng/mL tocotrienol-rich fraction or alpha-tocopherol, prior being exposed to 180 mM glutamate for 24 h [42]. Consistent with the results of Saito et al. (2010) [41], tocotrienol-rich fraction and alpha-tocopherol reduced lipid peroxidation and displayed comparable mitochondrial protective and cytoprotective capacities against glutamate-induced insults. In addition, flow cytometry analysis using annexin V-FITC, cell cycle analysis using RNase/PI assay and morphological observation using scanning electron microscopy collectively demonstrated the capacity of tocotrienol-rich fraction in restoring glutamate-induced apoptosis via promoting re-entrance of injured cells into S phase (DNA synthesis) and G_2_/M phase (DNA repair and recovery) of cell cycle [42]. Again, the underpinning alterations in gene expression and activity of components that orchestrate the cell cycle, particularly the cyclin-cyclin-dependent kinase (c-cdk) complex were not investigated in this study. Thus, a definitive conclusion could not be drawn on the molecular mechanism of tocotrienol to restore excitotoxicity in astrocytes.

Despite wide implication of the mevalonate pathway in the development of AD, only one study has investigated the effects of tocotrienols on Aβ load and cholesterol levels in neuronal cells on top of antioxidative effects [43]. In line with the results of the previous cell-based studies, Grimm et al. (2016) reported the reduction of ROS levels in SH-SY5Y human neuroblastoma cells by alpha-tocotrienol. Alpha-tocotrienol was shown to reduce total and free-cholesterol levels more profoundly than alpha-tocopherol in treated SH-SY5Y cells, measured by Amplex Red-based cholesterol assay [43]. The underlying molecular mechanism of this observation had been previously elucidated and reviewed by many research groups [29,44,45]. However, contradictory to previous findings on the reduction of Aβ protein by hypocholesterolemic drugs, an unfavorable effect of alpha-tocotrienol was reported, wherein Aβ protein levels were elevated in both normal and APP-overexpressing cells. Both β-secretase (APP to sAPPB and CTP99) and gamma-secretase (CTP99 to Aβ) activities were found to increase when cell membranes were purified and cultured with tocotrienol and in CTP99 transfected cells. Nonetheless, no changes were observed in gene transcription of β-secretase *BACE1* and components of gamma-secretase. BACE1 and nicastrin protein levels also remained unaltered. By evaluating Aβ degradation, the study showed that alpha-tocotrienol reduced Aβ40 degradation via a mechanism that involved IDE, a key enzyme in Aβ degradation [43]. It is worth noting that the authors did not investigate the formation and degradation of Aβ42, which plays a more profound role in the pathogenesis of AD. It is also unknown whether cell treatment with a lower alpha-tocotrienol concentration that has been reported to exert cytoprotective effects may alter Aβ levels in neuronal cells. Nevertheless, the findings of this study highlighted that tocotrienol treatment might not be beneficial to every patient with AD. Tocotrienol is most likely to be effective in hypercholesteraemic patients due to its ostensible cholesterol-lowering effects. In patients with normal cholesterol level, the amyloid potential of tocotrienol could be harmful. However, this speculation needs to be validated in future studies.

**Figure 1:**
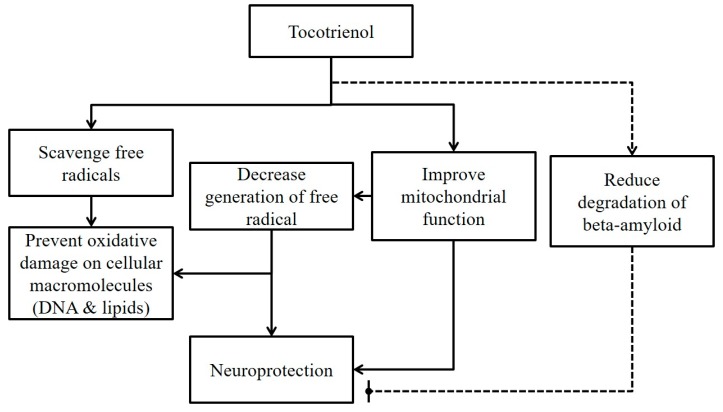
A summary of the current in vitro evidence of neuroprotective actions of tocotrienol. Legend: Solid line represents beneficial effects of tocotrienol on neurons. The dotted line presents potential adverse effects of tocotrienol on neurons.

The neuroprotective action of tocotrienol is summarized in Figure 1.

## 4. Evidence from Animal Studies

Animal studies pertaining to the effects of tocotrienol on neurodegeneration in animals focus mostly on the aspects of oxidative stress and mitochondrial function. Using male APPswe/PS1dE9 mice (a model of AD), Damahuri et al. (2016) showed that 200 mg/kg tocotrienol-rich fraction (tocotrienol-rich fraction; composition: 24% α-tocopherol, 27% α-tocotrienol, 4% β-tocotrienol, 32% γ-tocotrienol and 14% δ-tocotrienol) for six months suppressed erythrocytic superoxide dismutase activity but increased catalase activity. Alpha-tocopherol at the same dose could only lower the activity of superoxide dismutase. Comet assays revealed that both tocotrienol-rich fraction and alpha-tocopherol prevented DNA damage [46]. It should be noted that the untreated mice did not suffer from higher oxidative stress and DNA damage compared to the wild-type mice. Thus, it is a poor model to investigate the role of oxidative stress in the development of AD. Another limitation of this study is that the activity of antioxidants was measured in the blood, not the brain. Another study by Schloesser et al. (2015) showed that 369 mg/kg diet annatto tocotrienol (10% gamma-tocotrienol and 90% delta-tocotrienol) in cyclodextrins (providing 100 mg/kg tocotrienol in diet) for 24 weeks marginally increased the mRNA expression of superoxide dismutase 2, heme oxygenase 1 and gamma-glutamyl cysteinyl synthetase but did not affect expression of catalase and glutathione peroxidase 4 in the brain of aged Male C57BL/6J mice (15 months old) fed with high energy diet. Additional tests showed that the treatment improved mitochondrial function, by increasing membrane potential and ATP level. This was attributed to increased mitochondrial number, reflected by elevated mitochondrial transcription factor A (initiator of mitochondrial DNA duplication) expression. This study hinted the influence of tocotrienol on mitochondrial function [47].

Following this clue, two other studies further explored the effects of rice bran extract rich in tocotrienol (composition: 151.78 µg/g α-tocopherol, 53.55 µg/g α-tocotrienol, 0.53 µg/g β-tocotrienol and 236.48 µg/g γ-tocotrienol) on mitochondrial function using aged Young male Dunkin Hartley guinea pigs (50 and 150 mg/kg for 30 days) and Naval Medical Research Institute mice (340 mg/kg for 3 weeks) [48,49]. Rice bran extract improved the mitochondrial function of the supplemented animals to a varying degree, ranging from increased respiration control ratio due to decreased leakage in the electron transport chain in guinea pigs, to elevation of ATP levels and rejuvenation of complex I respiration in mice without increasing the respiration control ratio. These two studies agreed that the enhanced mitochondrial function was due to an increase in mitochondrial content, which in turn was a result of elevated mitochondrial abiogenesis (reflected by a rise in peroxisome proliferator-activated receptor gamma coactivator 1-alpha protein). A subtle difference was that mitochondrial fission (a rise in drp1 and fis 1 proteins) was attributed to enhanced mitochondrial repair and function in guinea pigs fed with rice bran extract, but mitochondrial fusion (a rise in mitofusin and opa proteins) was attributed to improvement in supplemented mice [48,49]. Species and dosage differences might explain some of the differences between these studies. It is noteworthy that rice bran extract contains other beneficial phytochemicals, such as gamma-oryzanol and polyunsaturated fatty acid. Thus, the interaction between these compounds and tocotrienol in improving neurological function of the animal could not be excluded.

These studies did not determine the phenotypic and behavior improvements caused by tocotrienol. Normal ageing animal models might lack the sensitivity to detect subtle behavioral changes. Tiwari et al. (2009) compared the efficacy of 21-day treatment of 50 and 100 mg/kg palm tocotrienol (a mixture of alpha-, beta- and gamma-tocotrienol; exact composition not mentioned) and 100 mg/kg alpha-tocopherol in Wistar rats treated with intracerebroventricular streptozotocin. Sub-diabetic doses of intracerebroventricular streptozotocin were previously shown to cause cognitive impairment in animals [50]. All treatment improved the performances of the rats in Morris water maze and elevated maze tests. The efficacy of palm tocotrienol was superior compared to alpha-tocopherol. The authors attributed these improvements to decreased acetylcholinesterase activity, nitrosative stress and lipid peroxidation, as well as increased antioxidant activity in the brain of the supplemented rats [51].

In a more recent study, Ibrahim et al. (2017) showed that the tocotrienol-rich fraction at 60 mg/kg/day for 10 months improved the ability of APPswe/PS1dE9 mice in recognizing novel objects. This was associated with decreased Aβ deposits in the brain, especially the cortex. Ex vivo examination of the same study showed that the tocotrienol-rich fraction reduced the formation of Aβ aggregates and oligomerization. However, this was not shown in the animal study [52].

## 5. Evidence from Human Studies

Four studies on the relationship between AD and tocotrienol were found, of which one was cross-sectional studies and three were prospective studies (Table 1). All studies measured circulating vitamin E levels using high-performance liquid chromatography. Most defined dementia based on Diagnostic and Statistical Manual of Mental Disorders (IV version), probable AD based on National Institute of Neurological and Communicative Disorders and Stroke-Alzheimer’s Disease and Related Disorders Association (NINCDS-ADRDA) criteria, and MCI based on Consensus Criteria for Amnestic MCI.

In a cross-sectional study involving 521 subjects from the AddNeuroMed Project (168 AD patients aged 74.7 ± 5.3 years, 166 MCI patients aged 75.8 ± 5.6 years, and 187 normal controls aged 77.4 ± 6.43 years), Mangialoasche et al. (2012) indicated that subjects with AD had significantly lower plasma levels of total vitamin E, total tocopherols, total tocotrienols and each vitamin E homologue compared to normal controls. Subjects with MCI shared a similar difference compared to normal controls, except that no difference in beta-tocotrienol level was found. In addition, the ratios of alpha-tocopherylquinone/alpha-tocopherol and 5-nitro-gamma-tocopherol/gamma-tocopherol were significantly higher in AD and MCI subjects. Both alpha-tocopherylquinone and 5-nitro-gamma-tocopherol are oxidation products of alpha-tocopherols and indicators of oxidative and nitrosative stress in the body. Using logistic regression models, the occurrence of MCI and AD could be predicted by vitamin E, its constituents and oxidation products. Subjects in the highest tertile of plasma total tocotrienols had an 8% probability of being diagnosed as having MCI (expressed in odds ratios (OR) and 95% CI) (OR MCI: 0.08 (CI: 0.02–0.26)), and 6% probability of being diagnosed as having AD (OR AD: 0.06 (CI: 0.02–0.21)) compared to subjects in the lowest tertile. Risk reduction for subjects in the highest tertile of total tocopherols (OR MCI: 0.15 (CI: 0.05–0.44); OR AD: 0.16 (CI: 0.05–0.46)) and vitamin E (OR MCI: 0.15 (CI: 0.05–0.42); OR AD: 0.15 (CI: 0.05–0.45)) were similar. Meanwhile, the probability of having AD or MCI increased multi-folds for subjects in the highest tertile of alpha-tocopherylquinone/alpha-tocopherol (OR MCI 4.43 (CI: 1.27–15.5); OR AD: 25.3 (CI: 6.8–93.7)) and 5-nitro-gamma-tocopherol levels (OR MCI 48.1 (CI: 12.7–182); OR AD: 11.0 (CI: 3.1–39.0)). The role of other lipid-soluble antioxidants, such as retinol and beta-carotene, in MCI and AD were also investigated. Like vitamin E, there was an inverse relationship between them and the occurrence of the disease [53]. Despite the significant relationship between oxidative stress markers and AD, the analysis of this study was not adjusted to the smoking status of the subjects. The levels of alpha-tocopherylquinone and 5-nitro-gamma-tocopherol were known to increase in smokers, and thereby potentially confounded the results of this study.

In a prospective study followed up for six years involving 232 dementia free-subjects (145 controls aged 84.6 ± 3.2 years and 57 cases aged 86.2 ± 2.8 years) from the Kungsholmen Project, subjects in the highest tertiles of total plasma vitamin E (HR: 0.55 (0.32–0.94)), total tocopherols (HR: 0.55 (0.32–0.94)), total tocotrienols (HR: 0.46 (0.23–0.92)) had 45%, 45% and 54% lower risk of developing AD compared to subjects in the lowest tertiles. The researchers reassessed the relationships, taking into consideration the potential confounding effects of cholesterol on circulating vitamin E levels. Beta-tocopherol, alpha-, beta-, gamma- and total tocotrienols were associated with a lower risk of AD in subjects with a cholesterol level below median but vitamin E level above the median. This study, although novel, involved a small number of very old subjects [54]. Other limitations included the collection of non-fasting blood samples, and the lack of consideration for the changes in dietary habit within the six years year of follow-up.

Similar results were obtained in the Finnish cohort of Cardiovascular Risk Factor, Aging and Dementia (CAIDE) study. A total of 140 subjects aged 64–70 years were followed up for 8.2 years. Beta-(absolute value) and gamma-tocotrienol level (with and without adjustment to cholesterol levels) were significantly lower in subjects who developed cognitive impairment. Subjects in the middle tertile of gamma-tocotrienol level had 27% (OR: 0.27 (CI: 0.10–0.78)) probability of developing cognitive impairment compared to those in the lowest tertile. In addition, subjects in the middle and highest tertile of 5-nitro-gamma-tocopherol/gamma-tocopherol ratio had a 3.4-fold probability and 2.9-fold probability of developing cognitive impairment. Taking cholesterol levels into account, higher gamma-tocotrienol was associated with lower risk of cognitive impairment (OR: 0.33 (CI: 0.10–1.06)) in subjects with total vitamin E level above median and cholesterol level below median. The authors acknowledged the small sample size and long duration of plasma sample storage for this study. They did not consider other nutrients present in vitamin E-rich food [55].

A prospective study from AddNeuroMed Project (81 subjects with AD aged 75.1 ± 5.7 years, 86 subjects with MCI aged 74.6 ± 5.2 years, and 86 control subjects aged 74.4 ± 5.5 years) attempted to increase predictability of AD and MCI by combining vitamin E levels and MRI results. Similar with the previous study, the absolute values of all vitamin E isomers were lower but the indices of vitamin E oxidative/nitrosative damage markers (alpha-tocopherylquinone and 5-nitro-gamma-tocopherol) were higher in the AD and MCI subjects compared to control. Adjusting for cholesterol rendered the difference of some homologues between cognitive-impaired and normal individuals not significant. The combination of vitamin E and MRI was better than MRI alone in predicting the occurrence of cognitive impairment (98% for AD, 91% for MCI, 95% for the conversion of MCI to AD after one year). Alpha-tocotrienol, gamma-tocotrienol, gamma-tocopherol and indices of vitamin E oxidative damage/nitrosative damage were the most relevant markers in predicting AD. Plasma vitamin E isomers were positively correlated with MRI regional cortical thickness and volumetric measures, and negatively correlated with ventricular volume. The inverse findings were found for vitamin E oxidative damage/nitrosative damage markers. The combination of a non-invasive (MRI) and minimally invasive (phlebotomy for blood collection) could complement the manual AD diagnostic criteria [56]. However, it was not known whether vitamin E were superior compared to other blood biomarkers in predicting AD. Furthermore, two major confounding factors of vitamin E levels, i.e., body mass index (BMI) and smoking status, were not adjusted in this study. Since vitamin E is lipophilic, potential sequestering effects by fat tissue was possible. If the confounding effect was present, it would weaken the relationship of vitamin E and cognitive impairment.

These observational studies are hypothesis-suggesting at best. They involved a relatively homogenous European population, so extrapolation to subjects of other ethnicities is difficult. Natural food contains a matrix of antioxidants which can contribute synergistically to the prevention of AD. Thus, the confounding effects of other nutrients on the relationship between tocotrienol and AD could not be excluded. The direct effects of tocotrienol supplementation on cognitive impairment cannot be validated due to the lack of clinical trials. On the other hand, these studies did consider every vitamin E isomer and its oxidative products. The follow-up period of the studies was reasonable.

## 6. A Comparison between the Role of Alpha-Tocopherol and Tocotrienol in Neuroprotection

Alpha-tocopherol is essential in maintaining normal neurological function in humans. The highly selective transporter of alpha-tocopherol, namely alpha-tocopherol transfer protein, is detected in the brain [57]. A loss-of-function mutation of the gene coding for alpha-tocopherol transfer protein will cause vitamin E deficiency ataxia [58]. Neurodegenerative diseases encompass not only AD, but also Parkinson’s disease, Huntington’s disease and amyotrophic lateral sclerosis. The accumulated evidence showed that alpha-tocopherol might have the potential in treating some of the conditions [59,60,61]. However, several meta-analyses disputed the efficacy of alpha-tocopherol supplementation in preventing and treating AD [62,63,64]. This is because a high level of alpha-tocopherol might decrease the bioavailability of other tocopherol isomers beneficial to the brain. Post-mortem brain examination revealed that high gamma-tocopherol was associated with less neuropathology associated with AD [65]. Moreover, at low levels of gamma-tocopherol, increased alpha-tocopherol was associated with high Aβ load in the brain [65].

In contrast, tocotrienols are not essential nutrients in our body. Apart from AD, studies on the effects of tocotrienol in treating other neurogenerative diseases are limited. Preclinical studies showed that tocotrienols prevented Parkinson-related toxicities in cultured neurons, as well as the loss of dopaminergic neurons in an animal model of Parkinson’s disease [66,67]. These effects were mediated by the oestrogen receptor-beta on the neurons [66,67]. However, the effects of tocotrienol on Huntington disease and amyotrophic lateral sclerosis have not been tested.

Both alpha-tocopherol and tocotrienols possess antioxidant activities, which mitigate oxidative damage in neurons. They also exert prominent cholesterol-lowering effects, which are associated with decreased Aβ production by reducing the stimulation of β- and γ-secretase activity [68]. Yet, the study by Grimm et al. (2016) revealed that both alpha-tocopherol and alpha-tocotrienol increased Aβ production independent of their cholesterol-lowering effects [43]. Therefore, vitamin E treatment might not improve the situation of all patients with neurodegenerative diseases, and non-responders to vitamin E treatment might exist [68].

The similarities and differences in the neuroprotective effects of alpha-tocopherol and tocotrienol are listed in Table 2.

## 7. Prospects for Future Research

Despite the potential benefits of tocotrienol on AD demonstrated by preclinical studies, there are several research gaps that have not been bridged. Firstly, both tocotrienol and statins are suppressors of distinct enzymes along the mevalonate pathway [69,70,71]. Several epidemiological studies have shown that the use of statins is linked to lower risk of AD and slower AD progression [72,73], which was attributed to its inhibitory effects on cholesterol and isoprenoid synthesis [15,74]. Since the biological effects of tocotrienol and statins on the mevalonate pathway are similar, it is reasonable that tocotrienol could improve cognitive function in a similar manner. However, no mechanistic studies have been performed to prove directly the involvement of mevalonate pathway in the neuroprotective action of tocotrienol by measuring its effects on HMGCR activity and mevalonate level in the neurons.

Secondly, no clinical trial has been conducted to show the efficacy of tocotrienol supplementation in preventing AD in the elderly. The current evidence is from four observational studies from a single research group. Since it is difficult to determine the causal relationship between variables in observational studies, the current evidence is suggestive at best. Only a well-planned clinical trial can resolve this issue. The optimum dose and circulating concentration of tocotrienol required for neuroprotection remain elusive at this moment. Extrapolation of the dose derived from animal studies could be difficult due to the difference among animal models used, ranging from healthy to genetically modified rodents with a tendency to develop AD.

Thirdly, oral bioavailability of tocotrienol is poor [75]. Its absorption and distribution to the circulation are hindered by the presence of alpha-tocopherol, the most abundant vitamin E in the food and the body [76]. Besides, previous studies showed poor distribution of tocotrienol in the brain upon oral administration [77,78]. Innovative methods should be developed to enhance the oral bioavailability of tocotrienol so that a sufficient amount could be delivered to the brain to exert its effects. High-dose tocotrienol should be used with caution, especially among patients with bleeding tendency or those taking anticoagulants because previous animal studies showed that it could prolong clotting and bleeding time [79,80].

Lastly, there are limited studies comparing the protective effects of alpha-tocopherol, individual tocotrienol isomers and natural tocotrienol mixtures directly in AD models. Both tocotrienols and alpha-tocopherol were shown to be protective against AD [41]. Although tocotrienols display better biological activities in vitro, alpha-tocopherol level is higher in the brain upon oral administration [77,78]. However, we could not conclude which formulation is the most efficacious against AD.

Continuous research is needed to overcome these limitations and understand the neuroprotective action of tocotrienol, which in turn could justify its use in the prevention of AD and cognitive impairment.

## 8. Conclusions

The current evidence suggests that tocotrienol is a potential neuroprotective agent which can prevent the development and hinder the progress of AD. Its abilities to reduce oxidative stress and promote cellular repair contribute to its beneficial effects on neurons. Despite the positive data from human epidemiological studies, there is no clinical trial to support the AD-preventing actions of tocotrienol. This research gap will have to be bridged by future researchers to establish tocotrienol as a neuroprotective agent.

## Figures and Tables

**Table 1:** Epidemiology studies on the relationship between tocotrienol and Alzheimer disease.

No.	Authors (Year)	Subjects	Tocotrienol Assessment	AD/MCI Assessment	Findings
1	Mangialasche et al. 2013 [56]	Prospective study. 81 with AD, 86 with MCI and 86 control subjects from AddNeuroMed Project. 1 year follow up	HPLC	Dementia: Diagnostic and Statistical manual of Mental Disorders (IV version)	MCI and AD patients had lower vitamin E (including tocotrienol) but higher vitamin E nitrosative and oxidative markers. The combination of vitamin E and MRI predicted the occurrence of cognitive impairment and the conversion of MCI to AD after 1 year better than MRI alone. Vitamin E, as well as its nitrosative and oxidative markers, were correlated with structural variation of the brain.
				Probable AD: National Institute of Neurological and Communicative Disorders and Stroke-Alzheimer’s Disease and Related Disorders Association (NINCDS-ADRDA) criteria.	
				MCI: Consensus criteria for amnestic MCI.	
2	Mangialasche et al. 2013 [55]	Prospective study. 140 subjects (aged 64–70 years) free from cognitive impairment from Cardiovascular Risk Factor, Aging and Dementia (CAIDE) study in Finland. Followed up for 8.2 years.	HPLC	Dementia: Diagnostic and Statistical manual of Mental Disorders (IV version)	Subjects who developed cognitive impairment had lower gamma- and beta-tocotrienol. Subjects with higher gamma-tocotrienol had lower risk to develop cognitive impairment. Subjects with higher gamma-tocopherol nitrosative marker had higher chances of developing cognitive impairment.
				AD: National Institute of Neurological and Communicative Disorders and Stroke-Alzheimer’s Disease and Related Disorders Association (NINCDS-ADRDA) criteria.	
				MCI: Mayo Clinic Research Centre Criteria.	
3	Mangialasche et al. 2012 [53]	Cross-sectional study. 521 subjects from AddNeuroMed Project: 168 AD (age: 74.7 ± 5.3 years), 166 MCI (age: 75.8 ± 5.6 years), 187 normal (age: 77.4 ± 6.43 years).	HPLC	Dementia: Diagnostic and Statistical Manual of Mental Disorders (IV version)	The levels of each vitamin E isomers and in total were significantly lower in AD and MCI subjects. They also had higher vitamin E nitrosative and oxidative markers. Diagnosis of MCI and AD were associated with reduced plasma level of total tocopherol, total tocotrienol and total vitamin E and increased vitamin E oxidative and nitrosative markers.
				Probable AD: National Institute of Neurological and Communicative Disorders and Stroke-Alzheimer’s Disease and Related Disorders Association (NINCDS-ADRDA) criteria.	
				MCI: Consensus criteria for amnestic MCI.	
4	Mangialasche et al. 2010 [54]	Prospective study. 232 dementia-free subjects from Kungsholmen Project aged > 80 years. Followed up for 6 years.	HPLC	Clinical and neuropsychological evaluation based on Diagnostic and Statistical Manual of Mental Disorders (III version)	Subjects with higher total vitamin E, total tocopherol and total tocotrienol level had a lower risk of developing AD. After adjusting for cholesterol level, beta-tocopherol, alpha-, beta-, gamma- and total tocotrienol were associated with a lower risk of AD in subjects with a cholesterol level below median but vitamin E level above the median.

**Table 2:** A comparison in neuroprotective effects between alpha-tocopherol and tocotrienol.

Biological Activity	Alpha-Tocopherol	Tocotrienol
Cytoprotective effect against glutamate toxicity [41]	Less efficient	More efficient
Scavenge ROS [41]	Yes	Yes
Reduce lipid peroxidation [41]	Yes	Yes
Neuroprotective effect in vivo [41]	More potent	Less potent
Reduce total and free-cholesterol levels [41]	Less efficient	More efficient
Elevate Aβ protein levels [43]	Yes	Yes
Increase amyloidogenic APP processing [43]	Yes	Yes
Decrease degradation of Aβ protein [43]	Yes	Yes
Decrease AD progression rate in human [62,63]	No significant changes	No significant changes
